# Alcohol Intake Thresholds Among Individuals With Steatotic Liver Disease

**DOI:** 10.1001/jamanetworkopen.2023.47548

**Published:** 2023-12-14

**Authors:** Yee Hui Yeo, Yixuan Zhu, Juan Pablo Arab, Wenjing Ni, Xiaoming Xu, Junping Shi, Jie Li

**Affiliations:** 1 Karsh Division of Gastroenterology and Hepatology, Department of Medicine, Cedars-Sinai Medical Center, Los Angeles, California; 2 Department of Infectious Diseases, Nanjing Drum Tower Hospital Clinical College of Nanjing Medical University, Nanjing, Jiangsu, China; 3 Division of Gastroenterology, Department of Medicine, Schulich School of Medicine, Western University and London Health Sciences Centre, London, Ontario, Canada; 4 Department of Epidemiology and Biostatistics, Schulich School of Medicine, Western University, London, Ontario, Canada; 5 Departamento de Gastroenterología, Escuela de Medicina, Pontificia Universidad Católica de Chile, Santiago, Chile; 6 Department of Infectious Diseases, Nanjing Drum Tower Hospital Clinical College of Traditional Chinese and Western Medicine, Nanjing University of Chinese Medicine, Nanjing, Jiangsu, China; 7 Department of Hepatology, the Affiliated Hospital and Institute of Hepatology and Metabolic Disease, Hangzhou Normal University, Hangzhou, Zhejiang, China; 8 Department of Infectious Diseases, Nanjing Drum Tower Hospital, Affiliated Hospital of Medical School, Nanjing University, Nanjing, Jiangsu, China

## Abstract

This cohort study aims to elucidate the dose-dependent association of alcohol use with progression of steatotic liver disease using a noninvasive, low-cost method.

## Introduction

Alcohol consumption is a risk factor for metabolic dysfunction, including insulin resistance and type 1 and type 2 diabetes.^1^ Alcohol exacerbates liver disease progression, leading to advanced fibrosis and cirrhosis. A recent update in nomenclature, steatotic liver disease (SLD), encompasses all fatty liver diseases confirmed by histological or radiological evidence, irrespective of etiology.^2^

While individuals with severe SLD should abstain from alcohol, little evidence exists for acceptable alcohol intake for patients with early stages of SLD who are disinclined toward complete abstinence. We aimed to elucidate the dose-dependent association of alcohol use with progression of SLD using a noninvasive, low-cost method.

## Methods

In this cohort study, we obtained data from the US National Health and Nutrition Examination Survey III (1988-1994) database, followed through December 31, 2019. The National Center for Health Statistics Ethics Review Board approved this survey, and documented informed consent was obtained from participants. Because the data are deidentified and publicly accessible, no additional review board approval was required (eMethods in Supplement 1). SLD was defined as ultrasonography-confirmed hepatic steatosis.^2^ An intermediate or high risk for advanced fibrosis was defined by a Fibrosis-4 index (FIB-4) score of 1.3 or higher. Race and ethnicity data collected through questionnaires were assessed to examine disparities. This study followed the Strengthening the STROBE reporting guideline.

Statistical analysis was performed between July and August 2023. Baseline characteristics were compared using χ^2^ tests for categorical variables and *t* tests for continuous variables. Multivariable Cox regression with restricted cubic splines was used to investigate nonlinear associations between alcohol use and mortality. All statistical analyses were conducted using R, version 4.1.2 (R Project for Statistical Computing). All tests were 2-tailed, with significance set at *P* < .05.

## Results

Of 2834 individuals with SLD (968 non-Hispanic White [34.2%]; 1467 male [51.8%]; prevalence rate, 36.2% [5016 of 13 856]), 20.8% (n = 591) had intermediate or high risk of advanced fibrosis, with male predominance and higher prevalence of metabolic disorders. During 66 299 person-years of follow-up, individuals at intermediate or high risk for advanced fibrosis had a mortality rate of 4342 per 100 000 persons compared with 1099 per 100 000 persons in the low-risk group (Table).

**Table.** Baseline Characteristics of the Overall Population of Individuals With Steatotic Liver Disease With 66 299 Person-Years of Follow-Up^a^

Characteristic	Total (N = 2834)	Low risk for advanced fibrosis (n = 2243)^b^	Intermediate or high risk for advanced fibrosis (n = 591)^b^	*P* value
Mortality rate	1608/100 000	1099/100 000	4342/100 000	NA
Follow-up time, median (IQR), y	26.3 (20.8-28.1)	26.7 (25.3-28.5)	18.4 (10.8-25.7)	<.001
Age, median (IQR), y	42 (31-58)	39 (29-50)	63 (55-69)	<.001
Sex, %				
Male	1467 (51.8)	1109 (49.4)	358 (60.6)	<.001
Female	1367 (48.2)	1134 (50.6)	233 (39.4)	<.001
Race and ethnicity, No. (%)				
Mexican American	1074 (37.9)	851 (37.9)	223 (37.7)	.60
Non-Hispanic Black	692 (24.4)	553 (24.7)	139 (23.5)	
Non-Hispanic White	968 (34.2)	765 (34.1)	203 (34.3)	
Other^c^	100 (3.5)	74 (3.3)	26 (4.4)	
Poverty income ratio <1, No. (%)	686 (26.6)	552 (27.1)	134 (24.7)	.26
High waist circumference, No. (%)^b^	1490 (54.1)	1135 (52)	355 (62)	<.001
Impaired fasting glucose, No. (%)^b^	928 (32.8)	660 (29.5)	268 (45.4)	<.001
Hypertension, No. (%)	1284 (45.3)	872 (38.9)	412 (69.7)	<.001
Hyperlipidemia, No. (%)^b^	1906 (68.9)	1499 (68.4)	407 (70.9)	.25
High triglycerides, No. (%)^b^	1252 (45.9)	984 (45.5)	268 (47.6)	.37
Low level of HDL, No. (%)^b^	1171 (43.5)	914 (42.8)	257 (46.1)	.16
No. of drinking days per year, median (IQR)	52 (24-156)	52 (24-156)	104 (48-303)	<.001
No. of drinks per day on drinking day, median (IQR)	2 (3-5)	3 (2-6)	2 (2-5)	.007
Alcohol consumption per day, median (IQR), g^d^	2.8 (0-14)	2.8 (0-12)	4 (0-20)	.002
Excessive drinking, No. (%)^e^	372 (13.1)	257 (11.5)	115 (19.5)	<.001
Heavy drinking, No. (%)^e^	132 (4.7)	63 (2.8)	47 (8)	<.001

Abbreviations: FIB-4, Fibrosis-4 index; HDL, high-density lipoprotein; NA, not applicable.

SI conversion factors: To convert fasting glucose level to millimoles per liter, multiply by 0.0555; triglycerides to millimoles per liter of triglycerides, multiply by 0.0113; and HDL to milligrams per liter of HDL, multiply by 0.1.

^a^ Continuous values are presented as median (IQR) values, and categorical variables are presented as numbers (percentages). All tests were 2-tailed with a significance level set at α = .05.

^b^ Low risk for advanced fibrosis was defined by an FIB-4 score of lower than 1.3; intermediate and high risk for advanced fibrosis was defined by an FIB-4 score 1.3 or higher; impaired fasting glucose was defined as a fasting glucose level of 100 mg/dL or higher; high waist circumference was defined as greater than 35 inches (88.9 cm) for women or greater than 40 inches (101.6 cm) for men; high triglyceride levels were defined as fasting triglyceride level of 150 mg/dL or higher; a low level of HDL was defined as less than 50 mg/dL for women or less than 40 mg/dL for men; hyperlipidemia was defined as a low level of HDL or a high level of triglycerides.

^c^ Included all Hispanic groups, regardless of race, who were not Mexican American and all non-Hispanic groups from racial groups other than Black or White.

^d^ The amount of alcohol consumed was reported in standard drinks (a drink was defined as 12 oz of beer, 4 oz of wine, or 1 oz of liquor) and converted to grams using a multiplication factor of 14.

^e^ Excessive drinking was defined as 20 g or more per day for women and 30 g or more per day for men; heavy drinking was defined as 50 g or more per day for women and 60 g or more per day for men.

After adjustment for demographic characteristics and metabolic variables, restricted cubic spline curves were used to identify a nonlinear association between alcohol consumption and mortality in the low-risk group (*P* = .001 for nonlinearity). Mortality risk exceeded an adjusted hazard ratio of 1.00 at a 7.4-g daily intake (hazard ratio, 0.99) (Figure, A). In the intermediate- or high-risk group, a linear association between daily alcohol consumption and increased risk of mortality was observed (*P* = .65 for nonlinearity) (Figure, B).

**Figure.**
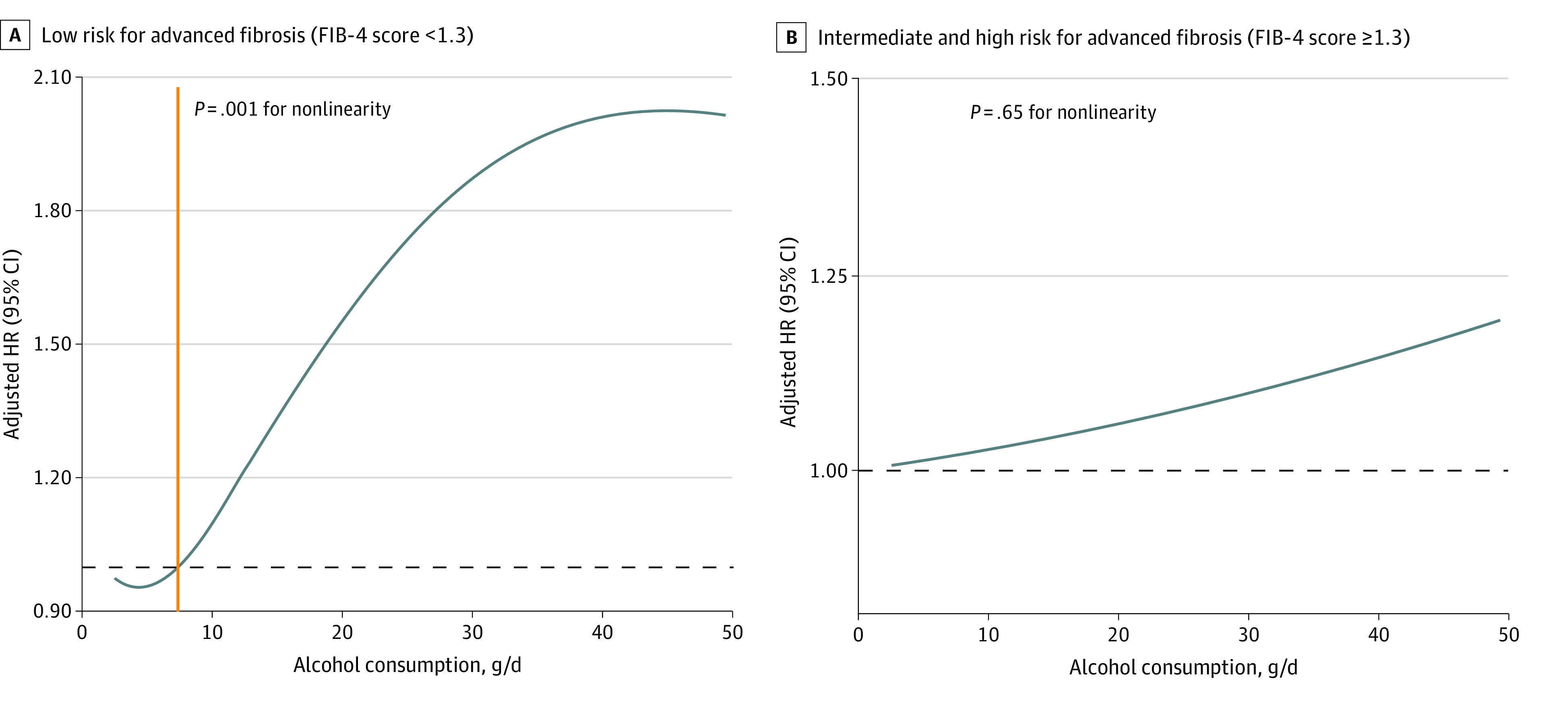
Association of Daily Alcohol Consumption With the Risk of Mortality for Individuals With Steatotic Liver Disease and at Different Stages of Fibrosis. Multivariable adjusted hazard ratios (HRs) for mortality according to alcohol consumption per day on a continuous scale were used. The model excluded patients with self-reported prior poor health, patients with comorbid-related conditions (heart failure, heart attack, stroke, or cancer), and nondrinkers. The solid blue lines are multivariable adjusted hazard ratios derived from restricted cubic spline regressions. The solid orange line indicates the daily alcohol consumption with the adjusted HR closest to 1 (dashed line). Analyses were adjusted for age, sex, race and ethnicity, poverty income ratio, hypertension, hyperlipidemia, impaired fasting glucose, and high waist circumference.

## Discussion

In this cohort study, the recommended level of alcohol consumption was less than 7.4 g/d for individuals with SLD at lower risk for advanced fibrosis, which equals half a 12-oz (336-g) beer or half a glass of wine.^3^ The finding aligns with previous research indicating higher risk of fibrosis progression among moderate drinkers.^4^ Notably, 7.4 g/d corresponds to half a standard US drinking unit and three-fourths of a traditional European drink, emphasizing modest ranges of acceptable alcohol consumption per individual.^5^ Individuals with SLD should be advised to maintain regular health monitoring and lifestyle management.^6^ Recent guidelines have recommended the FIB-4 score as a first-line assessment tool given its low cost, high accuracy, and noninvasiveness. To our knowledge, this study is the first to explore the utility of the FIB-4 score for individuals with SLD.

Our study is limited by relying on self-reported alcohol use and not considering drinking patterns. Individuals should not consume the weekly total in a single session. Additionally, all the variables were only available at baseline, with no tracking of alcohol intake changes during follow-up. Finally, individual risks may vary and require case-by-case discussion since the data are population based. In this cohort study, we proposed using the FIB-4 score to guide clinicians in advising patients with SLD who choose not to abstain completely from alcohol.

## Notes

Supplement 1.
eMethods.

eReferences.

Supplement 2.
Data Sharing Statement
