# Supplementary material for: Alcohol Intake Thresholds Among Individuals With Steatotic Liver Disease
Source: JAMA Netw Open. 2023 Dec 14;6(12):e2347548. doi: 10.1001/jamanetworkopen.2023.47548 (PMC10722338; doi:10.1001/jamanetworkopen.2023.47548)
Supplement: Supplement 1. — eMethods. eReferences. [file jamanetwopen-e2347548-s001.pdf]

Supplemental Online Content

Yeo YH, Zhu Y, Arab JP, et al. Intake thresholds among individuals with steatotic liver disease. *JAMA Netw Open*. 2023;6(12):e2347548.
doi:10.1001/jamanetworkopen.2023.47548

eMethods.
eReferences.

This supplemental material has been provided by the authors to give readers additional information about their work.

© 2023 Yeo YH et al. *JAMA Network Open.*

eMethods.

About NHANES III

The National Health and Nutrition Examination Survey (NHANES) is a periodic survey conducted by NCHS. The NHANES program began in the early 1960s and has been conducted as a series of surveys focusing on different population groups or health topics. In 1999, the survey became a continuous program that has a changing focus on a variety of health and nutrition measurements to meet emerging needs. The survey examines a nationally representative sample of about 5,000 persons each year. These persons are located in counties across the country, 15 of which are visited each year. *(https://www.cdc.gov/nchs/nhanes/index.htm)*

The third National Health and Nutrition Examination Survey (NHANES III), conducted from 1988 through 1994, was the seventh in a series of these surveys based on a complex, multi-stage sample plan. The NHANES interview includes demographic, socioeconomic, dietary, and health-related questions. The examination component consists of medical, dental, and physiological measurements, as well as laboratory tests administered by highly trained medical personnel. Institutional Review Board (IRB) approval and documented consent was obtained from participants. The data are de-identified and freely accessible to the public.

As the data are de-identified and freely accessible to the public, we downloaded the relevant data sets from the website *(https://wwwn.cdc.gov/nchs/nhanes/nhanes3/)*. In preparing a data set for analysis, other data files must be merged with either or both of these files to obtain many important analytic variables. All of the NHANES III public use data files are linked with the common survey participant identification number (SEQN). Merging information from multiple NHANES III data files using this variable ensures that the appropriate information for each survey participant is linked correctly. For each data file, SAS program code with standard variable names and labels is provided as separate text files on the CD-ROM that contains the data files. This SAS program code can be used to create a SAS data set from the data file.

The National Center for Health Statistics (NCHS) has linked data collected from several NCHS population surveys with death certificate records from the National Death Index (NDI). The public-use linked mortality

© 2023 Yeo YH et al. *JAMA Network Open.*

files (LMF) include a limited set of variables for adult participants only. The files provide mortality follow-up datafrom the date of survey participation through December 31, 2019. In the public-use LMF, each survey participant who is linkage eligible for mortality follow-up is assigned avital status code (0=Assumed alive, 1=Assumed deceased). The leading causes of death include diseases of the heart, malignant neoplasms, chronic lower respiratory diseases, accidents, cerebrovascular diseases, Alzheimer's disease, diabetes mellitus, influenza and pneumonia, nephritis, nephrotic syndrome and nephrosis (*National Center for Health Statistics Division of Analysis and Epidemiology. NHANES IIIPublic-use Linked Mortality Files, 2019. Hyattsville, Maryland. Available from:https://www.cdc.gov/nchs/data-linkage/mortality-public.htm. doi:10.15620/cdc:117141*).

Inclusion and exclusion criteria

SLD was defined as anyone with histology- or image-proven steatotic liver disease, irrespective of etiology [1]. Therefore, inclusion criteria for the study required evidence of hepatic steatosis. The ultrasound examinations were performed on 14,797 adults aged 20-74 years who were examined in NHANES III during 1988-1994. This study used a DVD-VHS Videocassette Recorder system to digitize videotapes of ultrasounds, and a board-certified radiologist trained three ultrasound readers to evaluate hepatic steatosis. Excluding missing and ungradable images, the following five ultrasonographic findings were used to evaluate the extent of steatosis: liver-to-kidney contrast, parenchymal brightening, deep beam attenuation, vessel walls, and gallbladder wall. Professionals classified hepatic steatosis into ordinary, mild, moderate, and severe. For the subsequent screening phase, individuals exhibiting mild to severe steatosis were selected as participants.

After excluding individuals with missing data on alcohol consumption and metabolic risk factors, our study included 2834 individuals with SLD for baseline analysis. In Multivariable Cox regression with restricted cubic splines, the models excluding patients with self-reported prior poor health, patients with comorbid related conditions (heart failure, heart attack, stroke, and cancer). Moreover, to avoid potential bias introduced by including individuals with zero alcohol use, possibly due to pre-existing health conditions leading to advised or chosen abstinence, we excluded non-drinkers from this study.

© 2023 Yeo YH et al. *JAMA Network Open.*

Study variable measurement

All participants completed a self-administered questionnaire, which included inquiries about alcohol use, medical history, and medical treatment. They also underwent a physical examination and provided blood samples for biochemical measurements. Hypertension was defined as a blood pressure value ≥140/90 mmHg or current use of antihypertensive drugs [2]. The diagnostic criteria for DM included self-reported medical history of DM, self-reported use of insulin or oral hypoglycemic agents, fasting plasma glucose (FPG) ≥126 mg/dL (7.0 mmol/L), 2-hour plasma glucose (2-h PG) ≥200 mg/dL (11.1 mmol/L) during oral glucose tolerance test (OGTT), or HbA1c ≥6.5% (48 mmol/mol) [3]. Impaired fasting glucose was defined as fasting glucose ≥100 mg/dL; High waist circumference was defined as >35 inches for women or >40 inches for men; High triglycerides were defined as fasting triglyceride level ≥150 mg/dL; Low HDL was defined as HDL <50 mg/dL for women or <40 mg/dL for men [4]; Hyperlipidemia was defined as low HDL or high triglycerides. The Fibrosis-4 (FIB-4) score was calculated as: (Age x AST) / (Platelets x √(ALT)). A FIB-4 score ≥1.3 was defined as intermediate or high risk for advanced fibrosis, while scores <1.3 were categorized as low risk [5].

Alcohol use

Alcohol consumption data were extracted from the universally administered alcohol use questionnaire. Individuals provided estimates of the average number of drinking days and the average number of drinks per typical drinking day in the past 12 months. The average number of drinks per day was calculated based on these values, and converted to grams using a multiplication factor of 14.

Statistical analysis

Our analysis began with the presentation of continuous variables as medians with interquartile range, while categorical variables were presented as counts with percentages. We developed multivariate Cox regression models to adjust for confounding factors (age, sex, ethnicity, poverty income ratio, hypertension, hyperlipidemia, impaired fasting glucose, high waist circumference). To investigate the dose-dependent relationship between alcohol use and mortality, we employed the theory of restricted cubic splines (RCS). RCS allows for the modeling of nonlinear relationships and provides a flexible method to describe the association between a predictor and an outcome. All analyses were performed using the R version 4.1.2.

© 2023 Yeo YH et al. *JAMA Network Open.*

eReference

1. Rinella ME, Lazarus JV, Ratziu V, et al. A multi-society Delphi consensus statement on new fatty liver disease nomenclature. *J Hepatol* 2023.
2. Williams, B.; Mancia, G.; Spiering, W.; et al. 2018 ESC/ESH Guidelines for the management of arterial hypertension: The Task Force for the management of arterial hypertension of the European Society of Cardiology and the European Society of Hypertension: The Task Force for the management of arterial hypertension of the European Society of Cardiology and the European Society of Hypertension. J Hypertens 2018;36:1953-2041, Oct, 2018.
3. Classification and Diagnosis of Diabetes: Standards of Medical Care in Diabetes-2020. Diabetes Care 2020;43:S14-s31, Jan, 2020.
4. Executive Summary of The Third Report of The National Cholesterol Education Program (NCEP) Expert Panel on Detection, Evaluation, And Treatment of High Blood Cholesterol In Adults (Adult Treatment Panel III). Jama 2001;285:2486-2497, May 16, 2001.
5. Sterling, R.K.; Lissen, E.; Clumeck, N.; et al. Development of a simple noninvasive index to predict significant fibrosis in patients with HIV/HCV coinfection. Hepatology 2006;43:1317-1325, Jun, 2006.

© 2023 Yeo YH et al. *JAMA Network Open.*
