# Supplementary material for: Alcohol Intake Thresholds Among Individuals With Steatotic Liver Disease
Source: JAMA Netw Open. 2023 Dec 14;6(12):e2347548. doi: 10.1001/jamanetworkopen.2023.47548 (PMC10722338; doi:10.1001/jamanetworkopen.2023.47548)
Supplement: Supplement 2. — Data Sharing Statement [file jamanetwopen-e2347548-s002.pdf]

Data Sharing Statement

Yeo. Alcohol Intake Thresholds Among Individuals With Steatotic Liver Disease. *JAMA Netw Open*. Published December 14, 2023. doi:10.1001/jamanetworkopen.2023.47548

Data
Data available: Yes
Data types: Deidentified participant data
How to access data: https://www.cdc.gov/nchs/nhanes/index.htm
When available: With publication

Supporting Documents
Document types: None

Additional Information
Who can access the data: Anyone requesting the data
Types of analyses: For any purpose
Mechanisms of data availability: With investigator support
